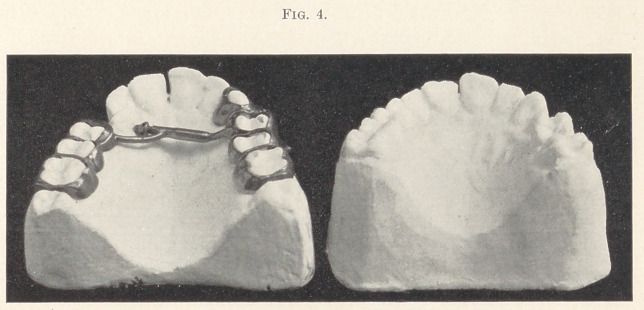# Some Æsthetic Considerations in the Treatment of Teeth in the Incisal Region

**Published:** 1901-11

**Authors:** B. Holly Smith

**Affiliations:** Baltimore, Md.


### International Dental Journal.

Vol. XXII. November, 1901. No. 11.

### Original Communications.[1]

### SOME ÆSTHETIC CONSIDERATIONS IN THE TREATMENT OF TEETH IN THE INCISAL REGION.[2]

BY B. HOLLY SMITH, M.D., D.D.S., BALTIMORE, MD.

Mr. President and Members of the Academy,—I believe that the suggestion that I should be here at this time was made by Dr. Andrews, and I count myself fortunate that my introduction to this society comes through one who is so highly regarded and honored, not only by its members, but by every one who knows of his work and worth; and as I come from a section where friendship means much, in that with us one's friends are often reckoned to do well because they are one's friends, I am emboldened to hope that the mantle of Dr. Andrews's friendship may cover any failure of mine to measure up to this opportunity.

It is not because I am under the impression that dentists as a whole are indifferent to æsthetic effect that I have determined to direct this discussion in the line indicated by the title of this paper, but because, by grouping many individual operations and subor-

[1] The editor and publishers are not responsible for the views of authors of papers published in this department, nor for any claim to novelty, or otherwise, that may be made by them. No papers will be received for this department that have appeared in any other journal published in the country.

[2] Read before the American Academy of Dental Science, Boston, May 1, 1901.

dinating them to their influence upon the general expression of the mouth and the pleasurable effect upon the eye of the beholder, I have conceived that an emphasis may be given to the necessity of protecting our patients from any show of art or apparent imperfection in nature. In a community such as this, where culture and refinement are the rule rather than the exception, it is to be presumed that the necessity for many of these suggestions is not apparent. If this be true, which I doubt not, let me ask you to allow me to be for the time being your apostle, and hope that my printed rather than spoken words may find such a field as I know exists elsewhere, thus giving, with your approval, an excuse for their having been written.

American dentistry, looked upon from the stand-point of the salvation of teeth, has made a record which has, perhaps, no equal in the annals of other countries; and for ingenuity and resourcefulness the American dentist in his particular field stands quite the peer of those surgeons who perform to-day so many wonderful feats in operations upon the brain and abdomen. It must be acknowledged, however, that while this record was being made many criticisms have been passed which were not solely the result of jealousy, but had some foundation in justice. I have always contended for and insisted upon professional authority. The professional man knows, and therefore his views have right of way. His patients are, or should be, as clay in the hands of the potter; for this reason I am intolerant of the plea that there is a demand by the patient for any work which is open to criticism. The dentist created the demand for a gaudy display of gold, and in so doing, unmindful of his high and holy mission as teacher of his fellow-men, has given opportunity for the outcropping of that barbaric love of glamour.

If you will allow me to divide my subject into different parts, I think we may approach its discussion with greater ease.

1. The use of gold for filling.
2. Porcelain, oxyphosphate, etc.
3. Crown- and bridge-work.
4. Shaping the natural teeth.
5. Orthodontia for patients of middle life.

### THE USE OF GOLD FOR FILLING.

It is easy to trace the influence of individual operators by the character of operations to be seen from the hands of their followers; and this is the more marked when it is studied from a sectional stand-point.

Varney and Webb wielded a wider influence than did the teachers of operative dentistry in the colleges, and to-day their followers may be found thickest around the scene of their late activities. Unfortunately, however, for the reputation of the leaders, those who adopt their methods do not always grasp the true intent and meaning of the teaching; they have seen their operations and try to copy them, but their efforts are not guided by their deft manipulation nor modified by their careful consideration of effect. The live coal from the altar has not been used; the true professional inspiration is too often missed in the absorption of mechanics. This is one of the inevitable disadvantages; where the teacher's contact with the scholar is so limited, he does not have an opportunity to root and ground him in his principles, to train him in the detail of the work, to impregnate him with his high ideals. I recall having witnessed at the hands of the late Dr. Webb a beautiful operation. The cavity was a mesio-proximal one in a right superior central incisor; the labial plate of enamel was without support from dentine almost to the full extent of the mesial third, and the mesio-incisal angle was damaged. In the operation this labial plate was protected with cement, the incisal extension was made about to the middle of the distal third,—the extension being chiefly at the expense of the lingual plate. The whole operation was beautifully performed and finished, leaving very little gold in sight. And yet in less than a month I had occasion to see from the hands of some who witnessed this clever demonstration operations which outraged every æsthetic sense, but which were attempted because of what they saw performed by Dr. Webb.

With this lesson so vividly in my mind, I can but look with apprehension upon the Western agitation of "extension for prevention." In the hands of Drs. Black, Johnson, and a few others such a wise and discriminating application of the theory may be made that no great violence to æsthetics will result; but such teaching will end inevitably in the mutilation of teeth and the production of an effect which would remind one of the door of a brass foundry flung wide open. I have had recent occasion to see from the hands of very skilful operators some cases of extension for prevention run mad, and it gives me the excuse for making this plea for a more conservative practice. When the lips are parted in merriment, when happiness, whose chief charm is its contagion, fills the mind and heart, no exhibition of dentistry should occur to mar the agreeable effect. With this in view, we would lay down the axiom that the labial surfaces of the incisors and cuspids should not, if possible to avoid it, be violated by the appearance of gold.

When proximal surfaces are to be filled, the teeth should be spaced with cotton from the lingual aspect, the linguo-proximal angle cut back slightly with chisels or enamel-trimmers, and the teeth filled preferably with non-cohesive gold, so that when normal position is resumed we have the slightest V opening towards the lingual surface. This teaching I know has suffered at the hands of its friends, but when intelligently applied it has stood the test fifty and sixty years, saved the teeth, and preserved the natural appearance. If the labial wall is deficient and such operation as above indicated would leave a space, the filling must be knuckled with cohesive gold, leaving, if possible, only a line of the material exposed. If the labial plate cannot be saved, porcelain or platinous gold should be used for its replacement. Whenever the incisal edge is to be extended, platinous gold should be used, as its effect is more agreeable to the eye than pure gold. As a rule, I should say that if any gold is to be exposed it must not be of the non-cohesive variety. I have had frequent occasion to replace gold fillings which produced the effect of a cavity in the tooth. Patients have complained that these fillings have been polished and repolished, but always remained unsightly, causing members of the family to accuse them of having a cavity in the tooth. Upon removing these fillings I have invariably found them to be non-cohesive gold. The effect, I think, is one of shadows. The gold surface has been left parallel with the opposing surface, the shadow of which, falling on the surface of the filling, makes it appear dark. With cohesive gold this filling can be restored with a slight contour, showing, it is true, a narrow line of gold, but even this is more agreeable than the cavity appearance. There is absolutely no excuse to-day for filling labial cavities with gold. It is not enough to demand of a filling-material to be used in these sites that it will stop the cavity; it must also be agreeable to the eye.

### PORCELAIN, OXYPHOSPHATE, ETC.

If gold be criticised and condemned as unfit for labial filling, what can be said of the ragged, irregular appearance, both in form and color, of some of the work from the hands of the followers of the so-called New Departure? Distance surely lends enchantment here. I have seen in some mouths certainly a half-dozen different-colored cements, not one of them resembling in shade the tooth it filled so imperfectly. There is opportunity for improvement along this line. A few of the manufacturers make several shades of powder; one, the Harvard, about twelve. Many of these, however, are unlike the color of any tooth I ever saw, thus leaving us to the presumption that they were intended to modify the white cement and make possible a good match to the shade of the tooth. By making these combinations and keeping sections as shade standards, with directions as to proportioning colors, etc., we might secure sufficient variety. Even with our best effort in this direction, however, cement should be used for temporary work only. If used for proximal work, it soon washes and wears, leaving ragged edges, which are both unsightly and uncomfortable.

Now and then we find samples of gutta-percha which seem to do fairly well both as to color and durability, but on the whole its use is limited to the protection of deep-seated cavities from thermal changes.

What, then, shall we use for filling-material in the incisal sites? There is nothing left but porcelain, and this is far from meeting every demand. It cannot be regarded as permanent work, used as it is to-day in the form of inlays baked in matrices taken from the cavity, and my experience teaches me that unless the adjustment is most carefully made they are liable to displacement or fracture. Improvement is going on constantly, however, both in furnaces and bodies, and every dentist should have access to a furnace and essay to do porcelain work of some kind. Failures may result at first, but success will ultimately come. Undercuts in both cavity and porcelain are essential. The inlay should have the glaze ground off the surface which is to be next to the cavity. When set with cement, a trace of the liquid without powder should be placed on this surface; by this we get an adhesion which is of great advantage. All this and many other things must be done, and yet the individual operations cannot be pronounced perfect, though the general effect is good.

When labial cavities the result of imperfections in the enamel are situated near the centre or in the incisal third of the labial surface, in filling much depends upon a nice marginal relation, and I do not think perfection can be so nearly approached with inlays baked in matrices as with porcelain ground from sections of English teeth. I have many of these inlays which have been standing for ten or twelve years, where the relation was so perfect before they were set that they were difficult to remove from the cavity when fitting them. It may be objected that this work requires skill and time. I am sure that this does not in any sense bar it, as with a little attention to detail the average dentist is quite equal to its performance, and the average patient quite willing to pay for the time. It has the advantage of offering opportunity to match the shade exactly, and can be polished until the eye will not detect the union between porcelain and tooth. This polishing must be done with a wheel of fine grit, though the danger of damaging the margin of the inlay is not so great as it is with the baked inlay.

It must be that this grinding of inlays has fallen into disuse because of supposed difficulties which I do not find to exist, and I only insist upon its revival because I am assured by results that it will repay any effort. Then it need not be so tedious. I have the rough outline of the inlay made in the laboratory from a section of English tooth and adapted approximately to a cavity obtained from an impression. It comes to me shellacked to a piece of orange-wood or match-stick. A little fitting and grinding is usually sufficient to get a perfect adaptation, and it is then set with cement.

To those who contend that permanence and not effect is to be sought after, and that this can most frequently be secured when no restrictions are placed on the character of materials used, let me say that I am as anxious as any that no departure from the thoroughness which has given to American dentistry its reputation should obtain. If we will bring to porcelain work the same painstaking attention to detail, the same ingenious resourcefulness, which we have shown with gold, we will develop a degree of permanence not yet accomplished. Again, we are to consider that as the years go by the practitioner is in more intimate touch with his patients, who realize more and more that visits should be made at short intervals. The renewal or resetting of porcelain inlays is not taxing to patient or operator.

### CROWN- AND BRIDGE-WORK.

Of all offences against good taste, the use of gold for the restoration of the crowns of incisors, cuspids, or first bicuspids is to my mind the last to be forgiven, and, fortunately for our credit, it is not frequently done. It is quite common, however, to collar roots and tip porcelain teeth so that the gold may be unsightly, and this is quite unnecessary. I am convinced that the collar used in Richmond crowns is an evil to be avoided. The gingival relation to the neck of a tooth is so susceptible to change, and pericemental disturbances are so likely to be set up by the slightest encroachment of this collar, that (except in rarely inviting cases) it should be abandoned entirely. Instead, the root should be shaped high lingually if possible, the lingual half rising as a pyramidal process, while the labial half is cut back under the gum so as to form a bevel, over which the cervical edge of the porcelain face rests. The gold cap should be fitted with contouring pliers to grasp this lingual heel and be burnished down over the labial bevel, thus protecting the root effectively from fracture, making a strong crown, and avoiding any show of gold or danger of injury.

When tipping porcelain facings with gold is necessary, it is a good plan to bevel the facings at the expense of the lingual edge and have only a line of gold to cover the labio-incisal edge, making the occlusion, if possible, upon the lingual portion of the facing, where the gold is thickest; this avoids almost entirely the appearance of gold.

When abutments are required on cuspid or bicuspid teeth for bridges, bars should be used, filling around them with gold in preference to crowning them. Where gold crowns are found in the mouth covering the teeth under consideration and serving as abutments to small bridges, the labial surface of these crowns may be cut out and porcelain inlays introduced, making a marked improvement in the effect without greatly threatening the security of the bridge. Indeed, it is surprising what can be done when one's practice is set in the direction of avoiding a show of gold, and that this is desirable, nay, necessary, is the main contention of your essayist.

### SHAPING THE NATURAL TEETH.

Abrasion of teeth as the effect of malocclusion is everywhere apparent in the mouths of people in middle life and old age. They have used their teeth vigorously, or by reason of the loss of some of the molars have made substitution of the other teeth for grinding. The shortening of the bite through the occlusal wear of the molars where they remain intact or by malpositions where only one is left almost invariably affects the relation of the teeth in the incisal region. Most commonly the superior incisors are forced by the inferior to occupy a malposition labially. This flaring of the incisal edges of these teeth exposes often rugged and uneven margins and gives to the mouth generally a very unsightly appearance. The enamel from the incisal edge frequently is gone entirely, and the lingual as well as the labial edge of enamel is exposed to view. Sometimes individual teeth are forced entirely out of line. Any radical remedy, of course, would involve the bridging and crowning or restoration of the normal occlusion, but much can be done to improve the appearance and increase the comfort by stoning and shaping the front teeth, restoring as far as possible normal shape and relation.

Where elongation in individual teeth has occurred, the entire incisal third may often be removed, cutting in stages and using chloride of zinc where sensitiveness is encountered; this to be followed by a constant use of bicarbonate of soda on the part of the patient. As a usual thing dentine should not be exposed in a young mouth, but my experience shows no trouble from such treatment as above suggested, care being taken that the surface stoned and shaped be highly polished and burnished. This is an essential. When this character of work first appealed to me I had complaints; then I used the emery fine grit. Now I use emery, rotten and Arkansas stone, followed by pumice and whiting, the latter carried by moose-hide; finally, the burnisher is used.

### ORTHODONTIA.

In the consideration of my last topic or division I have four cases to present. Irregularities difficult, indeed, apparently impossible from a remedial stand-point, often present. It is particularly embarrassing to have these passed on by those who should be, as we claim to be, well equipped for emergencies. However, if we will write on the first page of every new diary or engagement-book the pledge that with what knowledge and skill we possess we will wage war on disease and accident in whatever shape it may present, it may be an inspiration to us. For years I was under the impression, I think not an uncommon one, that the correction of irregularities after youth had lapsed into middle age was impracticable. This proposition with experience was outlived or out-practised by the help of the pledge.

The models which I present of some recent cases in practice may

Fig. 1.

Fig. 2.

Fig. 3.

Fig. 4.

serve to encourage the attempt at correction without regard to age. The first case, a woman aged about fifty, had for twenty years or more been a patient of a gentleman who ranks very high as a skilful operator and capable practitioner. She was told twenty years before these models were made that she was too old to have her teeth regulated. The malposition promised to increase with age, and now it gave to the whole face an unsightliness which was painful. The least observing of her friends deplored it. She sought relief from her dentist in vain. Such correction as is shown in the models was made in about six months. The marked improvement in the expression of the face and the cheerfulness of the patient is not to be appreciated by observing these, but exists nevertheless.

Case second, a woman of forty-three years, had consulted several dentists, but received no encouragement. The two centrals gave to the face a monstrous appearance, which should have appealed successfully for relief.

Case third, from a neighboring city, aged thirty-five, had sought relief in vain for ten years. The correction is not complete, the work having been interrupted by pregnancy. The models show what progress was made in five months.

Case fourth is shown with appliance; the patient is twenty-eight years of age. He had appliances in his possession made by different dentists. This appliance is the third which I have constructed for him, the other two having been ineffective. This was placed in position, the patient instructed in its working, and requested to call in a few days. He returned in about a week with the tooth in position. The alveolus shows evidence of fracture. Though several years have elapsed, no indication of the death of the pulp is to be seen,—indeed, it is known to be alive and well.

These models are not shown as anything unusual; they represent conditions which may exist in any clientele. The emphasis I desire to make is that they all had opportunity for previous relief, which was not rendered. The inference is that there are at least a few practitioners who are, to say the least, indifferent to their opportunities for doing good. Until they are converted let us not fail to preach. May we not seat hard and fast upon the shoulders of every one the absolute responsibility of the dentist, not alone for the salvation of the teeth, but for the preservation or restoration of the mouth to its greatest effectiveness as the most expressive feature of the face.

Much that I have said in this paper might be appropriately said before a class of students, and you may think it impertinent in me to present matter before this society, composed, as it is, of past masters in the science and art of dentistry, which contains no evidence of original research and smacks so much of triteness and truisms. I will accept with what grace and cheerfulness I can command this stricture if you will allow my main contention, *i.e.*, that whatever may be the status of our knowledge and cultivation, the objectionable practices which this paper is meant to criticise still exist in nearly every section of our country. Practitioners who should and do know better allow the character of their operations to be determined by the measure of toleration on the part of the patient, and not by a cultivated sense of æsthetics, trained and obligated from the professional stand-point to do the best that can be done. From the stand-point, then, of my hearers these comments may be out of place, but this society and its members can have no higher or more worthy mission than the quickening and awakening of the professional conscience and the stimulation of our professional brethren everywhere to place their practice upon an ideal plane.

## Figures and Tables

**Fig. 1. f1:**
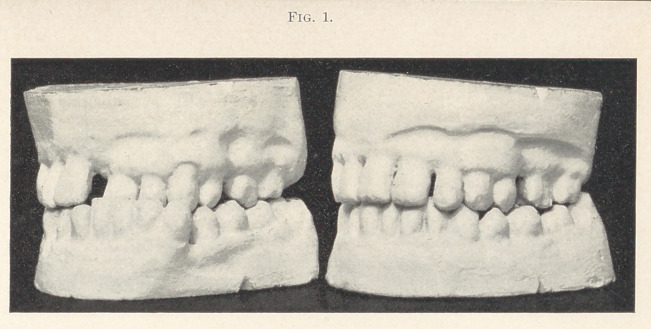


**Fig. 2. f2:**
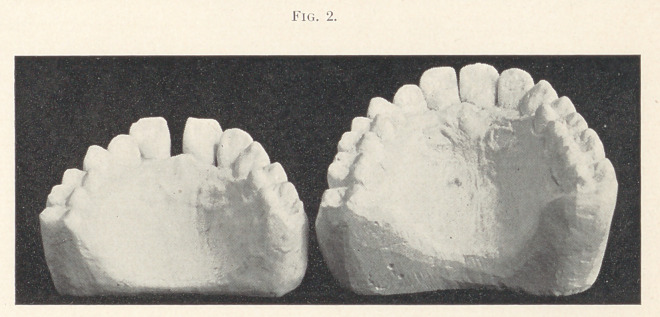


**Fig. 3. f3:**
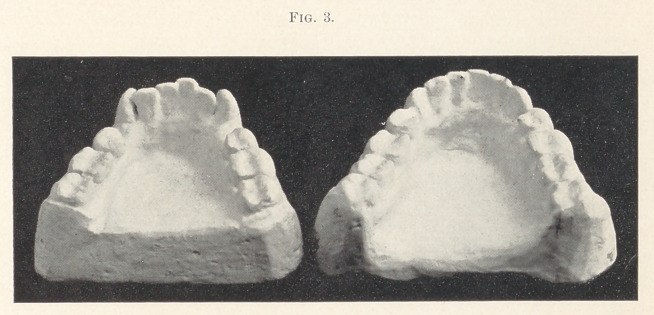


**Fig. 4. f4:**